# Supplementary material for: An automated model annotation system (AMAS) for SBML models
Source: Bioinformatics. 2023 Oct 26;39(11):btad658. doi: 10.1093/bioinformatics/btad658 (PMC10628433; doi:10.1093/bioinformatics/btad658)
Supplement: btad658_Supplementary_Data [file btad658_supplementary_data.pdf]

An Automated Model Annotation System (AMAS) for SBML Models: *Supplementary information*

Woosub Shin, John H. Gennari, Joseph L. Hellerstein, and Herbert M. Sauro

S1 Expressing ChEBI Identifiers as Chemical Formulas

In the ChEBI database, a single chemical species may have one *primary* identifier and several *secondary* identifiers. For example, as shown in Fig. S1, *water* can be described by CHEBI:15377, its primary identifier, but also by secondary identifiers such as CHEBI:13352 and CHEBI:5585. To solve the possible issue of identifiability, `AMAS` recommends species annotations only in primary identifiers.

We briefly describe considerations that `AMAS` makes for species with multiple annotations and/or multiple chemical formulas. Fig. S1 describes the existing ChEBI annotations of *M_h2o_e* in the `e_coli_core.xml` model. This species was annotated with 20 ChEBI identifiers including 6 primary identifiers, indicating protonation and deprotonation of the molecules. As a result, `AMAS` shortens existing or predicted ChEBI annotations into chemical formulas without hydrogen atoms when evaluating annotations or predicting reaction annotations using them. This would also reduce the computational cost of `AMAS`, as fewer unique molecules would be considered when making predictions. In our study, a total of 148,331 unique ChEBI primary identifiers could be mapped to 49,260 unique chemical formulas; the number was further reduced to 22,600 when the formulas were shortened.

Still, this may not be sufficient to completely remove the disparity between multiple chemical formulas for some reason. For example, after removing hydrogen atoms, *M_h2o_e* would still have O and [18O] due to isotopes. Another situation is when the string similarity yields multiple matches with the highest score. For example, for a query string 'ATP', the algorithm would be able to find a correct annotation of it, CHEBI:30616 with a formula of C10H12N5O13P3, but it may also detect CHEBI:15702 (terephthalic acid, C8H6O4), which has 'TPA' has its synonym. Since their cosine similarity will be both 1.0, both may be presented to users as likely candidates. However, it might be difficult to select a single chemical formula from the remaining ones; therefore, when predicting reaction annotations,

we use all of these remaining chemical formulas to find annotations of reactions as we discussed in the Methods section of the manuscript. Fortunately, we found our algorithm worked well in most cases and overall *exactness* to be high in our evaluation.

A related feature of our algorithm is that the hierarchy of ChEBI terms is disregarded when searching for species or reaction annotations, as long as the terms provide explicit chemical formulas. For example, CHEBI:17634 (D-glucose) is a parent term of CHEBI:4167 (D-glucopyranose), and both terms provide the same chemical formula of C6H12O6. However, the hierarchical relationship between the two terms are not used when `AMAS` computes the similarity between the query string and their synonyms. Similarly, once the species annotation has been transformed into the chemical formula, the difference between these two terms would vanish as their formulas would look identical.

One might be concerned that the transformation from ChEBI terms into (shortened) chemical formulas might result in incorrect predictions of annotations due to ignoring differences in chemical structures (isomers) or removing hydrogen atoms. However, even considering chemical formulas may be insufficient because of the occurrence of isomers. `AMAS` avoids many of these problems because it uses names, not chemical formulas to predict species annotations. `AMAS` does consider chemical formulas for reaction annotations, but transforms formulas by removing hydrogen atoms, an approach that might degrade the quality of predictions. For example, water (H2O, CHEBI:15377) and hydroxide (HO, CHEBI:16234) will be both mapped to O and this might cause including spurious annotations for a given query reaction. Fortunately, as we described in the result section, we found the quality of our recommendation to be robust; probably because most of the time, their annotations are predicted using a combination of multiple species and not using just a single species. In the future version of `AMAS`, we plan to introduce additional information from pathway databases so that the compatibility of predicted species and reactions can be checked; this way, false positive mappings would be detected and removed from predictions.

S2 Running `AMAS` as a Command-line Tool

`AMAS` has multiple scripts that can be run at the command line. Full details can be found in `readthedocs` at https://amas.readthedocs.io/en/latest/index.html.

S3 Statistics Related to `AMAS` Predictions

This section provides more detailed statistics about `AMAS` predictions.

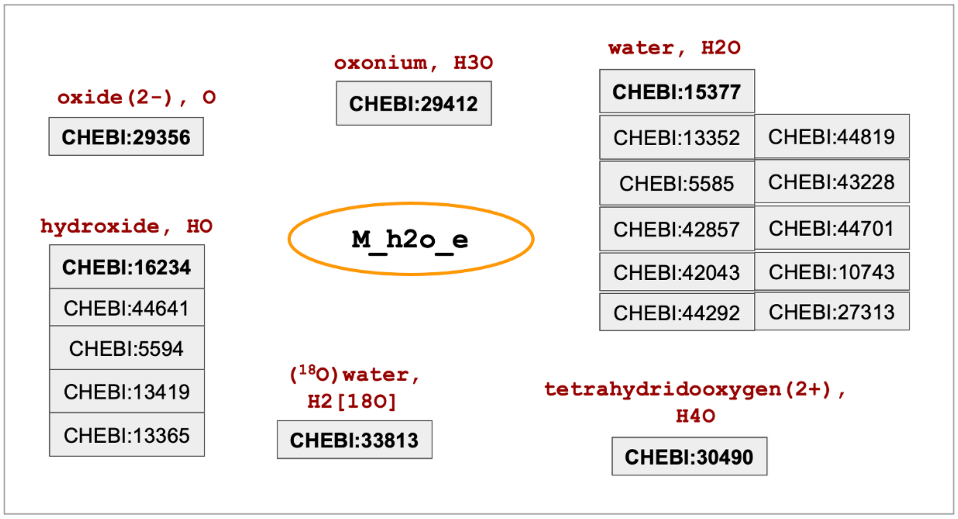

Fig. S1: Existing ChEBI annotations of *M_h2o_e*, a chemical species in the `e_coli_core.xml` model from the BiGG repository. A total of 20 unique identifiers were provided; they could be categorized into 6 unique groups, each with a primary identifier (bold) with a unique chemical formula. Even though the ID indicates *water (H2O)*, additional identifiers indicating protonation and deprotonation were also included in the existing annotations of the model.

From Fig. S2, we see the effect of filtering on the length of species names. The filters we consider result in an empty prediction set for query names that are shorter than 3 characters. But from the plots, we see that over 99% of the species names in BiGG and over 96% of the species in BioModels have a length of at least 3 characters. This suggests that there is only a modest decrease in *nonempty* if we filter query strings shorter than 3 characters.

Fig. S3 analyzes the match score cutoff in BiGG and BioModels for species and reactions. Recall that the match score cutoff is used by `AMAS` to filter the prediction set; an annotation is in the prediction set only if the match score for that annotation is at least as large as the match score cutoff. In BiGG, a match score cutoff of 0.6 results in *nonempty* being over 90% for both species and reactions. In BioModels with a match score cutoff of 0.6, *nonempty* is very large for species, almost 100%, and for reactions *nonempty* is over 70%. Of course, we know from the main text that prediction accuracy is larger with a larger match score cutoff.

These statistics motivate the iterative approach to annotations that we advocate in the main text. That is, the user should start with a large match score cutoff, say 0.9. If *nonempty* is too low, then decrease the match score, but scrutinize the additional annotations that are predicted.

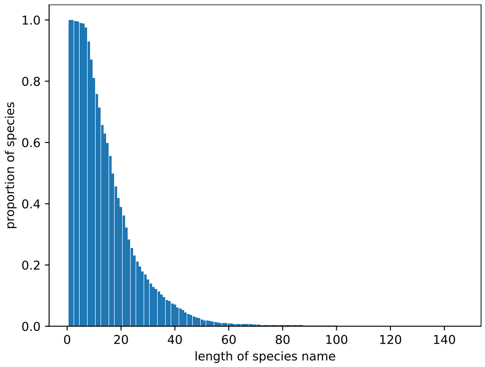

(a) BiGG

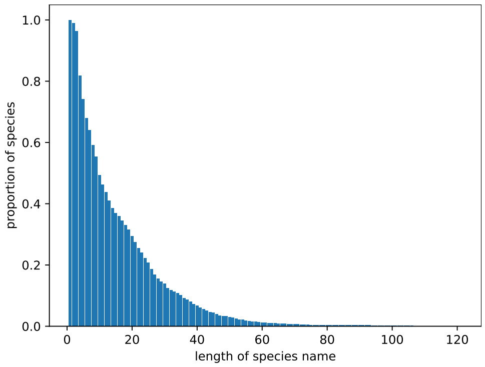

(b) BioModels

Fig. S2: Bar plots of name lengths.

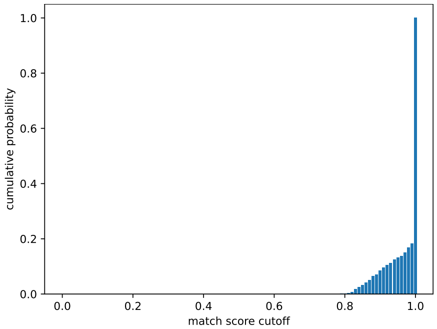

(a) BiGG species

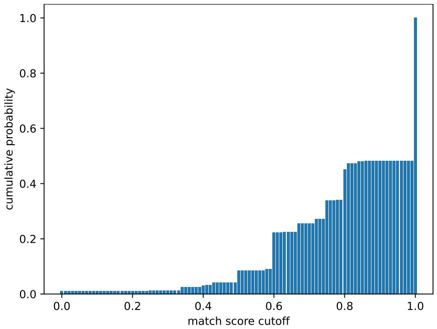

(b) BiGG Reactions

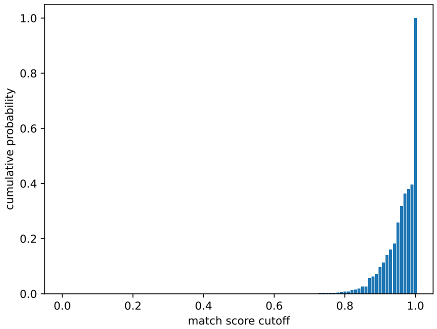

(c) BioModels species

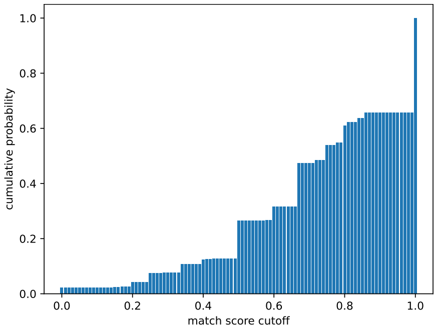

(d) BioModels reactions

Fig. S3: Match score CDF.

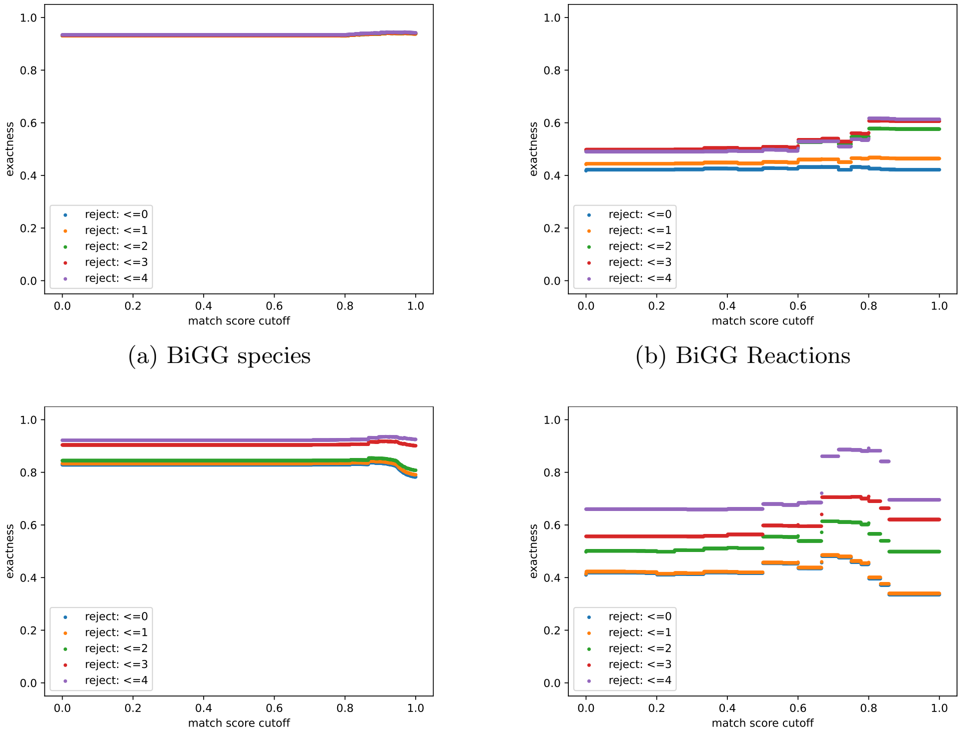

(a) BiGG species

(b) BiGG Reactions

(c) BioModels species

(d) BioModels reactions

Fig. S4: Exactness.

Fig. S4 displays details of *exactness* for different name length filters for species and reactions in BiGG and BioModels. As expected, the average *exactness* tends to increase with element filter and match score cutoff. Also we observe *exactness* always exceeds 0.33 (with a minimum of 0.335), indicating that the size of the prediction set averages at most three (and usually fewer) annotations.

S4 Levenshtein Distance

This section provides more details about the use of Levenshtein distance in `AMAS`.

At first glance, the string matching for `AMAS` match scores for species names sounds like polymer matching as is DNA, RNA, and proteins. However, the Needleman–Wunsch algorithm and related approaches assume much longer strings, and work best with a small alphabet and non-binary scoring for character mismatches. This led us to consider the Levenshtein distance, a calculation of the minimal number of insertions, deletions and replacements to make the two strings equal. We define the match score between the query species Q with string S and the reference species T with synonyms $S_1, \cdots, S_n$ as:

$$eScore(Q,T) = 1.0 - \min_{1 \le j \le n}(1.0, \frac{d(S,S_j)}{\max(|S|,|S_j|)}),$$

where $d(S, S_j)$ is the Levenshtein distance between the strings S and S_j. $|S|$ and $|S_j|$ represent the lengths of the strings.

We illustrate the formula with a simple example. Suppose that the query species has $S =$ "d_glucose" and that $T =$ CHEBI:17634. This ChEBI term has a single synonym "d-glucose", and so $S_1 =$"d-glucose". Note that $d(S, S_1) = 1$, since S and S_1 differ by a single character, and $\max(|S|, |S_1|) = 9$ as both have 9 characters. So, $eScore(Q, T) = 1.0 - min(1.0, \frac{1}{9}) \approx 0.89$.

eScore provides good accuracy for predicting annotations, but it is computationally intensive because of calculating Levenshtein distances. This is a serious problem since the computational complexity of predicting annotations is the computational complexity of calcuating the string match score multiplied by the number of reference species (over $140,000$) multiplied by the number of synonyms for reference species (up to 74 for each species).

S5: Calculating rScores

In our analysis of reaction match scores, we found that the number of shared species between the query reaction and the reference reaction was the most important factor to make reasonable predictions. In the current rScore formula, the numerator $(R^q \cdot R^r)$ represents this: the number of matches between the query and the reference elements. We tried a few other options for normalizing the numerator, such

as the number species in reference reactions or that in the query reaction, but we found that overall *accuracy*, *exactness*, and *nonempty* to be the highest with the current method. A related consideration is that different modeling approaches have been applied for models in BioModels and BiGG regarding their specification. Most BiGG reactions follow the standardized BiGG namespace, which tends to include all metabolites involved in the reactions, while many models in BioModels were manually created by model authors, often omitting some 'common' species in the specification. For example, in model BIOMD0000000408, reaction Jhyd_reaction indicates ATP hydrolysis and is specified as $ATP => ADP + P\_i$, not including H2O and proton. Our current method seems to handle both cases well by preserving the number of shared species between the query and the reference reactions.

Here, we provide a detailed example of calculating the rScore. Suppose we have the following reactions:

$$R^q : X \rightarrow Y$$

$$R^{r1} : X + Y \rightarrow Z$$

$$R^{r2} : X + Y \rightarrow W + U$$

$$R^{r3} : Y \rightarrow V$$

where X, Y, Z, W, U, V represent different species. According to the algorithm, (1) the first step is to find the maximum number of matches (M^q) between the query reaction R^q and the reference reactions R^{r1}, R^{r2}, and R^{r3}. Since R^{r1} and R^{r2} contain both X and Y, $M^q = 2$. (2) Next, the subset of reference reactions, $\mathcal{L}^q$, that have M^q (i.e., 2) matches with the query reaction is $\{R^{r1}, R^{r2}\}$. (3) Now, D is the smallest number in $\{R^{r_j} \cdot R^{r_j} | R^{r_j} \in \mathcal{L}^q\}$, so $D = R^{r1} \cdot R^{r1} = 3$. Then, the rScores are computed as below (rounded to two decimal places):

$$rScore_{R^{r1}} = \frac{2}{3} = 0.67$$

$$rScore_{R^{r2}} = \frac{2}{3} = 0.67$$

$$rScore_{R^{r3}} = \frac{1}{3} = 0.33$$

One alternative method we have considered is Jaccard Index, which is to divide the cardinality of intersection of the query element and the reference element by that of their union. The rScore can be computed using the formula:

$$rScore(R^q, R^r) = \frac{|R^q \cap R^r|}{|R^q \cup R^r|}$$

Therefore, the rScores for the above reference reactions are calculated as below:

$$rScore_{R^{r1}} = \frac{2}{3} = 0.67$$

$$rScore_{R^{r2}} = \frac{2}{4} = 0.50$$

$$rScore_{R^{r3}} = \frac{1}{3} = 0.33$$

Tab. S1 compares the results obtained by the current method and that by Jaccard Index. We found that Jaccard Index-based rScores produced overall higher *accuracy* and *exactness* than the current method but also yielded significantly smaller value of *nonempty*. Fig. S5 shows three quality metrics of the results obtained by Jaccard index, at various match score cutoffs, which shows the trend of overall small *nonempty* values. This seems to have been caused by the increased denominator (that is, the union of participants from the query reaction and the reference reaction), which makes the match score of many reactions smaller than that by the current method, reducing the discriminatory power by the match score cutoff. In the future version of `AMAS`, we are interested in exploring ways to adjust this approach so that it can be used as an alternative method for computing rScores.

Method	Accuracy	Exactness	Nonempty
Current	0.77	0.46	0.73
Jaccard	0.78	0.69	0.31

(a) BioModels

Method	Accuracy	Exactness	Nonempty
Current	0.69	0.43	0.90
Jaccard	0.89	0.69	0.36

(b) BiGG

Table S1: Mean *accuracy*, *exactness*, and *nonempty* obtained by the two different rScore methods. We applied MSSC Top with match score cutoff of 0.6.

S6: Optimizing Annotations

Predicted annotations can be further improved by exploiting the relationships between reactions and species, as depicted in Fig. 2.

Here, we provide more details on how `AMAS` optimizes predicted annotations. This is done in an iterative manner with `AMAS` updating species annotations based on reaction annotations. We note in passing that, once metadata of a species is updated in an SBML model, it will affect all reactions in the model that reference this species. This indicates annotation and match score of those reactions should also be considered. Therefore, `AMAS` updates reaction annotations based on the updated species annotations. The iteration continues until there is no improvement in the quality of the prediction or it reaches the maximum number of iteration

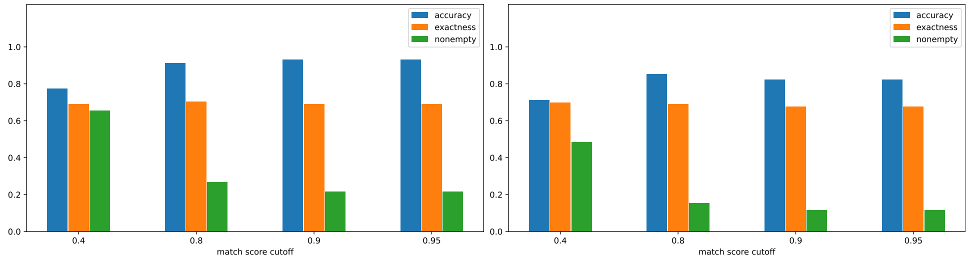

(a) BiGG (b) BioModels

Fig. S5: Quality metrics at different match score cutoffs, using the results obtained by Jaccard index. Compared with overall high *accuracy* and *exactness*, we can observe *nonempty* to be small.

(currently three). Prediction quality is quantified by the match score averaged over $rScore$ using updated species annotations. The details are:

1. For each query reaction in the model
   (a) Predict annotations for the query reaction using MSSC Top, and select an annotation.
   (b) Find the participants of the mapped reaction using its information in the reference database.
   (c) Match the participants of the mapped reaction with the participants of the query reaction.
   (d) Use the chemical formulas of the unmatched participants to find likely matches. Construct a list of pairings of a participant in the mapped reaction with a participant in the query reaction.
   (e) For each pair, assign the annotation of the participant of the mapped reaction to the query participant.
2. Reaction annotations are predicted again. Updated annotations for species are accepted if the resulting rScores increase.
3. If the updates are rejected or the match score does not increase or the iteration count is too large, stop. Otherwise, go to (1).

Due to computational complexity, the current algorithm uses just one mapped reaction with the highest match score, as well as comparing and updating when a single species is matched. We are planning to generalize this in the future algorithms using more computationally efficient ways such as that similar to the Expectation-maximization algorithm.

S7: Details of Evaluation of `AMAS`

There are some details as to how we extract annotations, especially in BioModels. For species, we used the ChEBI annotations under either *bqbiol:is* or *bqbiol:isVersionOf* qualifiers. Reactions are more complicated because BioModels provides few Rhea annotations. Rather, reactions are more frequently annotated with KEGG.Reaction or EC-number. We addressed this by mapping KEGG.Reaction and EC-number into Rhea terms using the cross-referencing information provided in the Rhea database.
